# Genetic polymorphisms of superoxide dismutases, catalase, and glutathione peroxidase in age-related cataract

**Published:** 2011-08-30

**Authors:** Yi Zhang, Lan Zhang, DongLin Sun, ZhiSheng Li, Lin Wang, Ping Liu

**Affiliations:** 1 Eye hospital, The First Affiliated Hospital, Harbin Medical University, Harbin, China; 2 Cardiovascular Medicine, The Fourth Affiliated Hospital, Harbin Medical University, Harbin, China; 3 Laboratory of Medical Genetics, Harbin Medical University, Harbin, China

## Abstract

**Purpose:**

The antioxidant enzymes pathway is considered the most important pathway involved in the repair of reactive oxygen species (ROS)–induced damage. Therefore, we investigate the possible association between polymorphisms of Cu/Zn superoxide dismutase (SOD1), catalase (CAT), and glutathione peroxidase (GPX) genes and age -related cataract development.

**Methods:**

The study included 415 cataract patients (121 patients with cortical, 109 with nuclear, 59 with posterior subcapsular, and 126 with mixed type) and 386 healthy control group of similar age. Genotyping of SOD1–251A/G, CAT–21A/T, and GPX1–198C/T was done by polymerase chain reaction and restriction fragment length polymorphism (PCR-RFLP) method. Differences in the frequencies were estimated using the χ^2^ test and risk was estimated with an unconditional logistic regression after adjusting for age and gender.

**Results:**

SOD1 G/G genotype frequency was significantly higher in cataract patients (p=0.012, OR=1.642, 95% CI=1.129–2.389). SOD1 A/A genotypes (p=0.001, OR=0.613, 95% CI=0.461–0.817) seem to have a protective role against cataract, and the G allele (p=0.001, OR=1.479, 95% CI=1.208–1.810) plays a dangeous effect against in the development of cataract. In CAT–21A/T and GPX1–198C/T polymorphisms, there were no significant differences in the variant homozygous frequencies in patients compared to controls (p=0.226, OR=1.358, 95% CI=0.839–2.199; p=0.521, OR=1.205, 95% CI=0.726–2.001, respectively). Stratification by the subtypes revealed that association between SOD polymorphism and cataract was in cortical and mixed type cataract. The genotype frequency of the GG and AA of SOD1–251A/G was significantly different in cortical and mixed type cataract group (p=0.031; OR: 1.805, 95% CI: 1.076–3.026; p=0.002; OR: 2.229, 95% CI: 1.364–3.645; p=0.026; OR: 0.608, 95% CI: 0.396–0.933; p=0.001; OR: 0.474, 95% CI: 0.305–0.734, respectively) compared to healthy controls.

**Conclusions:**

Results suggest that the G/G genotype of the SOD1–251A/G polymorphism may be associated with an increased risk of cataract. However, in CAT–21A/T and GPX1–198C/T polymorphisms, there were no significant differences in the variant homozygous frequencies in patients compared to controls.

## Introduction

One of the most common causes of blindness around the world is cataract, which is a multifactorial eye disease and a major cause of the loss of lens transparency in the aging population [1]. Oxidative stress is a major factor that often leads to cataract formation [2-4]. Oxidative stress is defined as a disturbance in the balance between the production of reactive oxygen species (ROS) and antioxidant defenses, including enzymatic and non-enzymatic systems [5,6].

ROS is mostly generated within the mitochondria in lens epithelium cells (LECs) and the superficial fiber cells, which are highly reactive. A certain level of ROS is crucial for the proper regulation of cell functions, such as intracellular signal, transcription activation, cell proliferation, inflammation, and apoptosis, but higher amounts of ROS are harmful to macromolecules [7]. In the lens, superoxide dismutase (SOD), catalase (CAT), and glutathione peroxidase (GPX), are some of the antioxidant enzymes that protect the organisms from oxidative damage [6,8,9]. SOD decomposes superoxide into hydrogen peroxide. CAT catalyzes the decomposition of hydrogen peroxide into water and oxygen, thereby preventing cell damage from high levels of ROS. GPXs are selenoproteins that reduce organic peroxides and hydrogen peroxide through the coupled oxidation of glutathione [10,11].

Genetic variations in the antioxidant genes coding for the SOD, CAT, and GPX enzymes may lead to decreased or impaired regulation of their enzymatic activity and alter ROS detoxification. Therefore, genetic variations among enzymes that protect the cell against ROS may modulate disease risk [12]. Due to the high interaction potentiality of ROS with genetic material, polymorphisms in genes coding for antioxidant enzymes may play an important role for inter-individual differences in maintaining the human genome’s integrity. Genetic polymorphisms in *SOD*, *CAT*, and *GPX* have been implicated in proneness to cancer and other diseases [13-15].

These potentially significant genetic variants related to oxidative stress have already been studied extensively, including single nucleotide polymorphisms (SNP) –251A/G of the *SOD1* gene (NCBI, refSNP ID: rs2070424), –21A/T in the promoter region of the *CAT* gene (NCBI, refSNP ID: rs7943316), and –198C/T of the *GPX1* gene (NCBI, refSNP ID: rs1050450). *SOD1*–251A/G, *CAT*–21A/T, and *GPX1*–198C/T polymorphisms are the most common and important of antioxidant enzymes. Most of these polymorphisms result in changes in the levels or the activities of these enzymes, which can lead to reduced protection against oxidative stress [12,15-19]. The effect of these variations on the lens has not yet been clarified, so we choose these candidate SNPs to study in our work.

The purpose of the present study was to evaluate the possible association between these enzyme’s genes and the risk of age-related cataract in the Chinese population. We analyzed *SOD1*–251A/G, *CAT*–21A/T, and *GPX1*–198C/T polymorphisms in patients with age-related cataract and otherwise healthy age-matched individuals in a control group. We hypothesized that polymorphic variations in these antioxidant genes modify the risk of age-related cataract, especially when oxidative stress is increased and non-enzymatic antioxidants are deficient.

## Methods

### Subjects

Patients with senile cataract were recruited from the Eye Hospital, the First Affiliated Hospital, Harbin Medical University, Harbin, China. This case-control study included a total of 415 patients with age-related cataract and 386 disease-free controls of Han Chinese. All 415 subjects with cataract had severe visual disturbances, and their corrected visual acuities were under 0.3, had an age-related cataract in at least one eye, and had no other eye abnormalities that could explain the vision loss. Cataract status was determined by the lens examination using a slit-lamp biomicroscope. Lens opacities were classified into cortical, nuclear, posterior subcapsular, and mixed type using the Lens Opacities Classification System II (LOCS II) [20]. We excluded patients with secondary cataract due to diabetes, trauma, steroid administration, and other causes. The sex and age-matched control subjects were collected from unrelated volunteers in the same clinic. Both groups belonged to the same ethnic group. The mean ages for the cataract patients and the controls were 67.17±6.92 years and 65.77±6.49 years, and of them 53.0% and 52.3% were men, respectively (Table 1). Informed consent was obtained from each subject before the study. The study was approved by the Bioethics Committee of the Harbin Medical University, China, and each patient gave written informed consent.

**Table 1:** Demographic data of the patients and controls.

**Demographic data**	**Cataract group**	**Control group**	**p-value**
Number of patients	415	386	
Gender	** **	** **	0.887
Male, n (%)	220 (53.0%)	202 (52.3%)	** **
Female, n (%)	195 (47.0%)	184 (47.7%)	** **
Age (years)	** **	** **	0.134
Mean±SD	67.17±6.92	65.77±6.49	** **

### Blood samples and DNA isolation

We collected 5 ml of venous blood into EDTA tubes from all of the patients and the controls. Immediately after collection, all blood was stored in aliquots at −80 °C until use. Genomic DNA was extracted from leukocytes using a Roche DNA purification kit (Roche Diagnostics GmbH, Mannheim, Germany) according to the manufacturer’s instructions.

### Genotyping of *SOD1*–251A/G

*SOD1* genotypes were detected using a multiplex PCR–RFLP method. An A→G in exon 10 (codon 399) was amplified to form undigested fragments of 570 bp using primers, described in Table 2. PCR conditions were 94 °C for 4 min, followed by 35 cycles of 94 °C for 50 s, 63 °C for 50 s, 72 °C for 50 s, and a final extension step at 72 °C for 7 min. The 570 bp PCR products were digested with MspI (MBI Fermentas, Burlington, CA) at 37 °C for 5 h and analyzed with 2% agarose gels. MspI digestion resulted in one fragment of 570 bp for wild-type (AA); two fragments of 369 and 201 bp for variant homozygous (GG); and three fragments of 570, 369, and 201 bp for heterozygous (AG).

**Table 2:** Primers for PCR amplification.

***SOD1*–251A/G primers**
5′-AGTACTGTCAACCACTAGCA-3′ (forward)
5′-CCAGTGTGCGGCCAATGATG-3′ (reverse)
***CAT*–21A/T primers**
5′-AATCAGAAGGCAGTCCTCCC-3′ (forward)
5′-TCGGGGAGCACAGAGTGTAC-3′ (reverse)
***GPX*–198 C/T primers**
5′-TGT GCC CCT ACG GTA CA-3′ (forward)
5′-CCA AAT GAC AAT GAC ACA GG-3′ (reverse)

### Genotyping of *CAT*–21A/T

*CAT* genotypes were detected using a multiplex PCR–RFLP method. An A→T in exon7 (codon 326) was amplified to form undigested fragments of 250 bp using primers, described in Table 2. PCR conditions were 94 °C for 4 min, followed by 35 cycles of 94 °C for 25 s, 62 °C for 25 s, 72 °C for 25 s, and a final extension step at 72 °C for 7 min. The 250 bp PCR products were digested with HInfI (MBI Fermentas) at 37 °C for 5 h and analyzed with 2.5% agarose gels. HInfI digestion resulted in two fragments of 177 and 73 bp for wild-type (AA); three fragments of 250, 177, 73 bp for heterozygous (AT); and one fragment of 250 bp for the variant homozygous (TT).

### Genotyping of *GPX*–198 C/T

GPX genotypes were detected using a multiplex PCR–RFLP method. An C→T in exon 10 (codon 198) was amplified to form undigested fragments of 338 bp using primers, described in Table 2. PCR conditions were 94 °C for 4 min, followed by 35 cycles of 94 °C for 35 s, 60 °C for 35 s, 72 °C for 35 s, and a final extension step at 72 °C for 7 min. The 338 bp PCR products were digested with ApaI (MBI Fermentas) at 37 °C for 5 h and analyzed with 2% agarose gels. ApaI digestion resulted in one fragment of 338 bp for variant homozygous (Leu/Leu); two fragments of 257 and 81 bp for wild-type (Pro/Pro); and three fragments of 338, 257, and 81 bp for heterozygous (Pro/Leu).

### Statistical analysis

Statistical analyses were performed with SPSS for Windows (version 17.0; SPSS Inc., Chicago, IL). Differences between the means of the two continuous variables were evaluated by the Student’s *t*-test. χ^2^ or Fischer’s exact test (two-sided) was used to compare the gender distribution, test the association between the genotypes and alleles in relation to the cases and controls, and test for deviation of the genotype distribution from Hardy - Weinberg equilibrium (HWE). The odds ratio (OR) and 95% confidence intervals (CIs) were calculated to estimate the strength of the association between polymorphism genotype alleles and patients and controls. Mean±SD are presented for continuous variables and p values of <0.05 were considered statistically significant.

## Results

There were no significant differences for sex, age, and ethnicity, which suggested that cataract patients' data are comparable with controls (see Table 1). Three genotypes were determined for all cataract cases and controls. For all polymorphisms, the more common allele was considered the reference genotype, whereas the less common allele was examined as the variant. The distributions of the *SOD1*-251A/G, *CAT*-21A/T, and *GPX*-198 C/T genotypes were in accordance with the Hardye Weinberg Equilibrium among the patients (p=0.187, p=0.265, p=0.825, respectively) and the controls (p=0.106, p=0.531, p=0.746, respectively). The frequencies of the genotypes and alleles of *SOD1*–251A/G, *CAT*–21A/T, and *GPX1*–198C/T polymorphisms in the age-related cataract and control groups are shown in Table 3.

**Figure 1:**
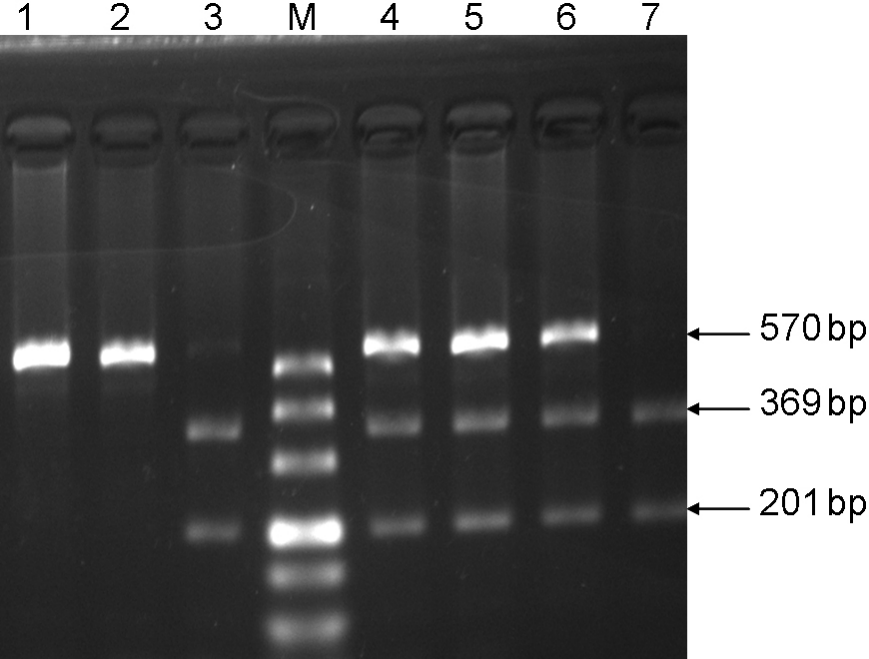
PCR analysis of *SOD1* gene polymorphism. One fragment of 570 bp for *SOD1* wild-type (AA), two fragments of 369 and 201 bp for variant homozygous (GG); and three fragments of 570, 369, and 201 bp for heterozygous (AG). Columns show the following: M column, DL500 DNA marker; 1 and 2 columns, *SOD1* wild-type (AA); 3 and 7 columns, *SOD1* heterozygous (AG); 4, 5, and 6 columns, *SOD1* variant homozygous (GG).

As shown in Figure 1, the analysis of the polymorphisms located at *SOD1* codon 399 in the cataract group showed that 138 (33.3%) were wild-type for the A/A genotype, 86 (20.7%) were variant homozygous for the G/G genotype, and 191 (46.0%) were heterozygous for the A/G genotype. There was a significant difference between the case and control groups in the *SOD1* G/G genotype (p=0.012). The statistical analysis revealed a possible dangeous effect of the *SOD1* G/G genotype (OR=1.642, 95% CI=1.129–2.389) in the development of cataract and a possible protective effect of the A/A genotype (OR=0.613, 95% CI=0.461–0.817) in controls. When compared to healthy controls, the allele frequency of the G allele of *SOD1*–251A/G was significantly different in the cataract group (p=0.001, OR=1.479, 95% CI=1.208–1.810).

However, no statistically significant differences were observed in the alleles or in the genotype frequencies of the *CAT*–21A/T, and *GPX1*–198C/T gene polymorphisms between the control group and the patients with age-related cataract (Table 3). In addition, there were no significant differences between the case and control groups in the *CAT* T/T genotype (p=0.226, OR=1.358, 95% CI=0.839–2.199). When compared to healthy controls, the allele frequency of the T allele of *CAT*–21A/T was not significantly different in the cataract group (p=0.123, OR=1.187, 95% CI=0.956–1.474). There was no significant difference between the case and control groups in the *GPX1* T/T genotype (p=0.521, OR=1.205, 95% CI=0.726–2.001). When compared to healthy controls, the allele frequency of the T allele of *GPX1*–198C/T was not significantly different in the cataract group (p=0.135, OR=1.183, 95% CI=0.952–1.471).

**Table 3:** Polymorphisms in antioxidant genes *SOD1*-251A/G, *CAT*–21A/T, *GPX*–198 C/T, and risk of cataract development.

**Genotype**	**Patients (%)**	**Controls (%)**	**OR (95% CI)**	**p-value**
***SOD1*–251A/G**				
AA	138 (33.3)	173 (44.8)	0.613 (0.461–0.817)	0.001
AG	191 (46.0)	160 (41.5)	1.204 (0.910–1.593)	0.200
GG	86 (20.7)	53 (13.7)	1.642 (1.129–2.389)	0.012
A allele frequency	0.563	0.655	** **	** **
G allele frequency	0.437	0.345	1.479 (1.208–1.810)	0.001
***CAT*-21A/T**				
AA	204 (49.2)	207 (53.6)	0.836 (0.633–1.104)	0.229
AT	167 (40.2)	148 (38.3)	1.083 (0.815–1.438)	0.613
TT	44 (10.6)	31 (8.1)	1.358 (0.839–2.199)	0.226
A allele frequency	0.693	0.728	** **	** **
T allele frequency	0.307	0.272	1.187 (0.956–1.474)	0.123
***GPX*-198 C/T**				
CC	201 (48.4)	208 (53.9)	0.804 (0.609–1.061)	0.137
CT	177 (42.7)	149 (38.6)	1.183 (0.892–1.569)	0.250
TT	37 (8.9)	29 (7.5)	1.205 (0.726–2.001)	0.521
C allele frequency	0.698	0.732		
T allele frequency	0.302	0.268	1.183 (0.952–1.471)	0.135

Table 4 shows the distribution of genotype frequencies of *SOD1*-251A/G, *CAT*-21A/T, and *GPX*-198 C/T polymorphisms in healthy controls and cataract patients after stratifying by the cataract subtypes. When compared to healthy controls, the genotype frequency of the GG of *SOD1*-251A/G was significantly different in cortical and mixed type cataract group (p=0.031; OR: 1.805, 95% CI: 1.076–3.026; p=0.002; OR: 2.229, 95% CI: 1.364–3.645, respectively), and the genotype frequency of the AA of *SOD1*-251A/G was significantly different in cortical and mixed type cataract group (p=0.026; OR: 0.608, 95% CI: 0.396–0.933; p=0.001; OR: 0.474, 95% CI: 0.305–0.734, respectively). However, the results for cortical cataract were no longer significant after Bonferroni correction (Bonferroni corrected p<0.017).

**Table 4:** Distribution of genotype frequencies of *SOD1*-251A/G, *CAT*-21A/T, and *GPX*-198 C/T polymorphisms in controls and patients with different cataract subtypes.

** **	**Cataract subtypes**				
**Genotype**	**Control n (%)**	**Cortical n (%) n=121**	**Nuclear n (%) n=109**	**PSCC n (%) n=59**	**Mixed n (%) n=126**
***SOD1*–251A/G**					
AA	173 (44.8)	40 (33.1)^a^	41 (37.6)	22 (37.3)	35 (27.8)^c^
AG	160 (41.5)	54 (44.6)	51 (46.8)	28 (47.5)	58 (46.0)
GG	53 (13.7)	27 (22.3)^b^	17 (15.6)	9 (15.2)	33 (26.2)^d^
***CAT*-21A/T**					
AA	207 (53.6)	62 (51.2)	55 (50.5)	29 (49.2)	58 (46.0)
AT	148 (38.3)	47 (38.9)	43 (39.4)	23 (39.0)	54 (42.9)
TT	31 (8.1)	12 (9.9)	11 (10.1)	7 (11.8)	14 (11.1)
***GPX*-198 C/T**					
CC	208 (53.9)	61 (50.4)	52 (47.7)	29 (49.1)	59 (46.8)
CT	149 (38.6)	52 (43.0)	49 (45.0)	25 (42.4)	51 (40.5)
TT	29 (7.5)	8(6.6)	8 (7.3)	5 (8.5)	16 (12.7)

PSCC, posterior subcapsular cataract. ^a^p=0.026; OR: 0.608, 95% CI: 0.396–0.933. ^b^p=0.031; OR: 1.805, 95% CI: 1.076–3.026. ^c^p=0.001; OR: 0.474, 95% CI: 0.305–0.734. ^d^p=0.002; OR: 2.229, 95% CI: 1.364–3.645.

Investigation of the genotype frequencies both in patients and controls revealed that there was a significant difference between frequencies for *SOD1*-251GG genotype in patients with cortical (22.3%) or mixed (26.2%) cataract and healthy controls (13.7%). There was a significant difference between frequencies for *SOD1*-251AA genotype in patients with cortical (33.1%) or mixed (27.8%) cataract and healthy controls (44.8%). The statistical analysis revealed that *SOD1*-251GG genotype may have a dangeous effect to the development of cataract, and *SOD1*-251AA genotype may have a protective effect against the development of cataract. No statistically significant difference was found for the genotypic and allelic distributions of the polymorphisms in *CAT*-21A/T, and *GPX*-198 C/T between controls and patients even after stratifying by the cataract subtypes. Haplotypes with frequencies are shown in Table 5. In age-related cataract patients, the frequencies of AAC, GAC were significant differences than those of controls (p=0.002, p=0.016), and the frequencies of AAT, ATC, ATT, GTC, GAT, and GTT were no significant differences than those of control (p=0.94, p=0.421, p=0.698, p=0.764, p=0.607, and p=0.082, respectively).

**Table 5:** Haplotype frequencies of *SOD1*-251A/G, *CAT*-21A/T, and *GPX*-198 C/T in controls and patients.

***SOD1*–251**	***CAT*-21**	***GPX*-198**	**Frequency**	**Patients**	**Controls**	**p-value**	**OR (95% CI)**
A	A	C	0.409	0.360	0.465	0.003	0.648 (0.488–0.860)
A	A	T	0.090	0.090	0.090	0.94	0.982 (0.605–1.593)
A	T	C	0.072	0.080	0.065	0.421	1.247 (0.728–2.139)
A	T	T	0.034	0.030	0.035	0.698	0.859 (0.399–1.852)
G	A	C	0.134	0.160	0.105	0.016	1.665 (1.095–2.532)
G	A	T	0.075	0.080	0.070	0.607	1.149 (0.677–1.949)
G	T	C	0.097	0.095	0.100	0.764	0.931 (0.583–1.485)
G	T	T	0.089	0.105	0.070	0.082	1.577 (0.956–2.602)

## Discussion

In this study, we analyzed the association between *SOD1*–251A/G, *CAT*–21A/T, and *GPX1*–198C/T polymorphisms with the risk of cataract in the Chinese population since these polymorphisms may change the enzymes’ antioxidant capacity and subsequently lead to synergistic effects with cataract induced by oxidative damage. In our experiments, we found that the *SOD1*–251A/G polymorphisms were associated with an increased risk of cataract, and we found no statistically significant association in *CAT*–21A/T and *GPX1*–198C/T polymorphisms between the controls and patients. These findings support the hypothesis that genetic variations in antioxidant defense may modify the risk of cataract among individuals with increased oxidative stress or decreased antioxidant capacity.

Defects in antioxidant pathways are connected to several different types of diseases, including diabetes, age-related diseases and cancer [12,13,21]. Antioxidant enzymes can catalytically remove free radicals and other reactive species. Among the antioxidant enzymes, SOD is considered to be important in the oxidant defense mechanism, as it is involved in the first line of defense [4]. The *SOD1* gene is located on chromosome 21p22.11, an A/G sequence variant at codon 251 that has been studied most frequently [17,18]. The *CAT* gene is located on chromosome 11p13.31 and contains 13 exons. There is a common polymorphism in the promoter region of the *CAT* gene consists of A to T at codon 21 [14,16]. The *GPX1* gene is located on chromosome 3p21.3. Several variants of *GPX1* have been described, and one affecting codon 198 that results in a C to T change has been studied most frequently [18,19]. Previous studies examining the association between ROS and various diseases have revealed that excessive oxidative stress or decreased antioxidant activity can cause several pathologic states [12,13,21,22].

To the best of our knowledge, no studies have investigated the role of antioxidant gene *SOD1*–251A/G, *CAT*–21A/T, and *GPX1*–198C/T polymorphisms for patients having cataract. Recent studies reported important associations of the *SOD1*–251A/G polymorphism with a reduced antioxidant capacity [15,18]. SOD is the major enzyme to scavenge the superoxide radical in all eye tissues. The studies of SOD activity in animal models and in humans with cataract have reported varying levels of red blood cell SOD activity in patients with different types of cataract. Researchers have also found that the level of SOD activity to be significantly lower in mature cataractous lenses than in clear lenses [4,23,24].

The lens is often under the threat of oxidative stress. ROS-mediated oxidative damage has been shown in the pathogenesis of cataract. ROS initiate lipid peroxidation leading to increased production of lipid peroxides in various forms of cataract. Mitochondrial production of superoxide increases with age. Superoxide may diffuse throughout the lens via the lens membranes. This process could contribute to elevated H_2_O_2_ in the nucleus of older lenses [25]. In normal conditions, LECs use several strategies to maintain ROS at low levels to protect lipids, proteins, and nucleic acids. These strategies include activation of the ROS scavenger enzymes such as SOD, CAT, and GPX, and nonenzymatic scavengers such as reduced GSH, ascorbate, cysteine, and vitamin E [26]. However, these ROS scavengers diminish with aging, placing the lens at risk for oxidative damage and cataract. A ‘‘vicious cycle’’ of decreased antioxidant activity and excess of ROS production may be established within cells, which may lead to a loss of the mitochondrial membrane potential, DNA damage and the release of cytochrome c, resulting in cell apoptosis and death [27,28]. The increased oxidative damage, decreased capacity of antioxidant enzymes, and apoptosis or death of LECs may cause a “vicious cycle” of oxidative stress that possibly contributes to the onset of cataract [24,29-31]. The SOD1 level was still represented 90% of the SOD activity in lens [32]. In this study, the polymorphisms of *SOD1*–251A/G may diminish the capacity to scavenge superoxide radical in lens, the superfluous radicals may contribute to the development of cataract.

Most studies have suggested that the polymorphisms of *CAT*–21A/T and *GPX1*–198C/T reduce their antioxidant capacity, these genes polymorphisms are risk factor for different kinds of diseases [14,16,18,19]. *CAT* and *GPX* are important components of cell defense against oxidative stress, but we did not find any association between these enzymes’ polymorphisms with the risk of cataract in our study. We propose several potential explanations for the lack of a relationship between the two enzymes’ polymorphisms and the risk of cataract in our study. First, cataract is a multifactorial disease and the pathogenesis is complicated. Specifically, it can be caused by exposure over time to a large number of physiologic signals that come through aqueous humor such as hydrogen peroxide, oxygen, and UV light, producing oxidative damage in the lenses of several mammalian species. The toxic effects of the ROS are generally controlled in the lens and aqueous humor by antioxidant enzymes and nonenzymatic scavengers [33-36]. Second, the exposure and interaction of other genes participating in antioxidant recognition, repair and cell cycle regulation may have altered the effect of *CAT* and *GPX* polymorphisms. Although multiple environmental factors are involved in the pathogenesis of cataract, many studies also can proved that genetic factors are also largely responsible for the development of lens opacity [37-40]. Haplotypes are thought to carry information about possibly unobserved causal variants in the region [41]. In the frequencies of the haplotypes, GAC were higher in patients compared with healthy controls, suggesting that this haplotype may increase the risk of age-related cataract. Furthermore, we found the haplotype frequencies of AAC were lower in patients compared with healthy controls. This result might imply a protective role for this haplotype of AAC in combination of age-related cataract in Chinese Han population.

In conclusion, this report is the first to suggest an association between the polymorphisms of antioxidant enzyme genes with cataract in the Chinese population. However, there is a need for other ethnicities and nationalities to confirm our findings, and to fully examine the possible relationship between antioxidant enzyme genes’ polymorphisms with cataract. These data emphasize that the importance of antioxidant enzyme genes’ polymorphisms and antioxidant enzymes as pharmacological targets to reduce ROS production may provide a strategy to prevent or slow the progression of age-related cataract formation.
